# Rare variants of small effect size in neuronal excitability genes influence clinical outcome in Japanese cases of *SCN1A* truncation-positive Dravet syndrome

**DOI:** 10.1371/journal.pone.0180485

**Published:** 2017-07-07

**Authors:** Michael F. Hammer, Atsushi Ishii, Laurel Johnstone, Alexander Tchourbanov, Branden Lau, Ryan Sprissler, Brian Hallmark, Miao Zhang, Jin Zhou, Joseph Watkins, Shinichi Hirose

**Affiliations:** 1 ARL Division of Biotechnology, University of Arizona, Tucson, AZ, United States of America; 2 Neurology Department, University of Arizona, Tucson, AZ United States of America; 3 Department of Pediatrics, School of Medicine and Central Research Institute for the Molecular Pathogeneses of Epilepsy, Fukuoka University, Fukuoka, Japan; 4 Interdisciplinary Program in Statistics, University of Arizona, Tucson, AZ United States of America; 5 College of Public Health, University of Arizona, Tucson, AZ United States of America; 6 Department of Mathematics, University of Arizona, Tucson, AZ United States of America; University of Pennsylvania, UNITED STATES

## Abstract

Dravet syndrome (DS) is a rare, devastating form of childhood epilepsy that is often associated with mutations in the voltage-gated sodium channel gene, *SCN1A*. There is considerable variability in expressivity within families, as well as among individuals carrying the same primary mutation, suggesting that clinical outcome is modulated by variants at other genes. To identify modifier gene variants that contribute to clinical outcome, we sequenced the exomes of 22 individuals at both ends of a phenotype distribution (i.e., mild and severe cognitive condition). We controlled for variation associated with different mutation types by limiting inclusion to individuals with a *de novo* truncation mutation resulting in *SCN1A* haploinsufficiency. We performed tests aimed at identifying 1) single common variants that are enriched in either phenotypic group, 2) sets of common or rare variants aggregated in and around genes associated with clinical outcome, and 3) rare variants in 237 candidate genes associated with neuronal excitability. While our power to identify enrichment of a common variant in either phenotypic group is limited as a result of the rarity of mild phenotypes in individuals with *SCN1A* truncation variants, our top candidates did not map to functional regions of genes, or in genes that are known to be associated with neurological pathways. In contrast, we found a statistically-significant excess of rare variants predicted to be damaging and of small effect size in genes associated with neuronal excitability in severely affected individuals. A *KCNQ2* variant previously associated with benign neonatal seizures is present in 3 of 12 individuals in the severe category. To compare our results with the healthy population, we performed a similar analysis on whole exome sequencing data from 70 Japanese individuals in the 1000 genomes project. Interestingly, the frequency of rare damaging variants in the same set of neuronal excitability genes in healthy individuals is nearly as high as in severely affected individuals. Rather than a single common gene/variant modifying clinical outcome in *SCN1A*-related epilepsies, our results point to the cumulative effect of rare variants with little to no measurable phenotypic effect (i.e., typical genetic background) unless present in combination with a disease-causing truncation mutation in *SCN1A*.

## Introduction

An important problem in medical genetics is identifying factors that influence the clinical outcome of Mendelian disease. Patients that carry the same disease-causing variant may develop a severe form of the disease, a mild form, or show no symptoms at all. Modifier genes represent one factor that may explain the extent of clinical variability in such cases. While studies in mice have been successful in identifying genes that modify the phenotypic expression of a mutant gene [1–3], the search for modifier genes in humans remains challenging. This is due to the fact that human populations carry genetic variation at many genes that could influence clinical outcome of monogenic disease, and variants at modifier genes may have no affect on the phenotype of individuals when not present in combination with the primary gene variant responsible for the disease [4].

Epilepsy disorders caused by sodium channel mutations represent a disease phenotype that may be strongly influenced by modifier genes. Truncation mutations in the voltage-gated sodium channel gene, *SCN1A*, usually result in Dravet Syndrome (DS)—a haploinsufficiency syndrome that includes severe progressive seizures and impaired cognition [5]. Most affected individuals are sporadic cases without affected family members; however, there are known examples of families in which an *SCN1A* truncation mutation is transmitted through multiple generations, with some members exhibiting typical DS, and others with milder epilepsy [6, 7]. Similarly, *SCN1A* missense variants often segregate in families in which there are members with DS, milder forms of epilepsy, and unaffected carriers [8–11]. Studies in mice have mapped several modifiers of *SCN1A*, many of which turn out to be genes coding for other ion channels [3, 12, 13].

The advent of next-generation sequencing technology has led to new approaches to identify modifier genes. A good example is the use of exome sequencing to identify a rare genetic modifier of clinical outcome in cystic fibrosis, which is commonly caused by variants in the cystic fibrosis transmembrane conductance regulator (*CFTR*). Emond et al. [14] sequenced the exomes of individuals at both ends of a phenotype distribution and identified *DCTN4* as a modifier of chronic *Pseudomonas aeruginosa* infection in cystic fibrosis. Because the frequency of alleles that contribute to the trait are enriched in one or both phenotypic extremes, a modest sample size can potentially be used to identify novel candidate genes and/or alleles.

In this study, we employ an extreme phenotype approach to search for genetic modifiers in a cohort of Japanese patients with *SCN1A* truncation-related epilepsy. We identified 22 patients with either mild or severe clinical expression as determined by cognitive and motor skills. We then sequenced the exomes of these patients and searched for common variants that were enriched in either the mild or severe phenotypic classes. We also performed rare variant burden tests to look for phenotypic associations with aggregates of variants across the exome. While we did not identify a single major modifier locus, our results suggest that rare pathogenic variants of small effect size in genes associated with neuronal excitability may tip the balance toward mild or severe clinical outcome in children with *SCN1A* truncation mutations.

## Material and methods

### Patients

We previously described a cohort of 285 patients with *SCN1A*-positive Dravet Syndrome [15] for which we had detailed clinical data that was collected by pediatric neurologists between September 2014 and September 2015. Referring physicians classified 176 of these 285 DS patients, as well as 12 other epilepsy patients with *SCN1A* variants, in one of five categories (normal, border, mild, moderate and severe) on the basis of intellectual and developmental quotient (IQ and DQ) assessment tests (Tanaka-Binet, WISC-IV, Enjoji Scale of Infant Analytical Development, Kyoto Scale of Psychological Development 2001, Kinder Infant Development Scale, and Tsumori-Inage Infant Mental tests), which varied by age of child. Five categories correspond to the following scores: IQ/DQ ≥85 normal, 70∼84 border, 50∼69 mild, 25∼49 moderate, ≤24 severe. It was also noted as to whether patients were bedridden/wheelchair bound, or whether they could walk and/or run. For this study we chose patients that: 1) were heterozygous for a truncation variant in *SCN1A* (nonsense, microdeletion, and frameshift), and 2) were classified in the opposite end of the phenotypic spectrum. We chose 12 of the most severely affected patients—those that were classified as severe in the shortest times after seizure onset (age range 57–150 months), and 12 of the most mildly affected patients—those that remained mild (i.e., normal, border or mild) for the longest period of time after seizure onset (age range 68–267 months). The parents of each of these patients provided signed informed consent using a protocol approved by the Ethics Review Committee of Fukuoka University, and all research was approved by the Institutional Review Board of the University of Arizona. When we resurveyed physicians in 2016, two mild patients progressed to moderate and severe phenotypes. The patient with severe phenotype qualified for inclusion in our severe group, and the patient with intermediate phenotype was excluded from further analysis. A possible limitation of this study is the small sample of patients with mild phenotype (see below).

### Exome sequencing

Whole exome sequencing was performed by array capture of 50 Mb of exome target sequence using the Agilent SureSelectXT Human All Exon V5 enrichment kit followed by paired-end sequencing (100 bases each read) on an Illumina HiSeq 2500. Sequences were trimmed with trimmomatic, v.0.32 (Bolger et al., 2014) and then aligned to the human genome (GRCh37) using Burrows-Wheeler Aligner, v.0.7.9a (Li & Durbin, 2010). Base quality recalibration, indel realignment, and calling of SNVs and small indels were performed using the Genome Analysis Toolkit, v.3.3–0, as previously described (McKenna et al., 2010). Variants were annotated using SnpEff v. 3.4 (Cingolani et al., 2012) with gene annotations made against Ensembl release 73. Previously known variants were annotated with their allele frequencies from the 1000 Genomes Project (www.1000genomes.org/data), the NHLBI GO Exome Sequencing Project (ESP) 6,500 samples release (http://evs.gs.washington.edu/evs/), and the Exome Aggregation Consortium (ExAC) release 0.3 (ftp://ftp.broadinstitute.org/pub/ExAC_release/).

### Filtering variants

The mean coverage over the exon target regions was between 46- and 89-fold for the 22 samples, with ~81–95% of RefSeq exonic base positions covered at least 20-fold. Genotypes were recorded at 460,528 sites, both inside and outside the target regions, after the removal of sites where more than 2 individuals had missing genotypes. We searched the 237 candidate genes associated with neurological excitability listed in Klassen et al. [16] (**S1 Table**) for variants at ≤1% in large public databases of exome sequences (i.e., the ExAC browser). Our coverage for this set of neurotransmitter receptor (n = 88), ion channel (n = 142) and other candidate genes (n = 7) was between 46-fold and 90-fold for the 22 samples, with ~81–95% of RefSeq exonic base positions covered at least 20-fold.

To represent the normal Japanese population, we downloaded 70 whole exome BAM files from the 1000 Genomes Project JPT population [17] and performed variant calling on these files using the same methods applied to our patient samples. After reviewing coverage over the 237 candidate genes described above, we chose a subset of 22 JPT samples to use in joint variant calling with our patient samples. This subset was chosen to minimize missing genotypes at the sites of the moderate or high-impact variants used in the rare variant analysis. The mean coverage over the target regions of the candidate genes ranged from 80- to 103-fold with ~88–93% of RefSeq exonic base positions covered at least 20-fold.

### Data analysis

We used two approaches to identify variants associated with clinical outcome. In the first approach, we focused on the genotypes of common (i.e., MAF >1%) variants and performed Fisher exact tests on genotype counts in the mild or severe phenotypic categories. In the second approach, we search for common and/or rare variants aggregated in and around genes throughout the genome in our exome sequence data. As described in Lee et al. [18], we adaptively combined a burden test and SKAT (described in SNP-set Sequence Kernel Association Test, Wu et al. [19]) to perform gene-based association tests on the WES data to assess the joint effects of multiple SNPs in a region on a binary outcome phenotypes (mild or severe). Adjusting for four covariates, age of seizure onset, time since onset (to present for mild, to first diagnosis of severe for severe), gender and motor delay, we obtain parameter estimates and residuals for the null model, which assumes there is no genetic association between genetic variables and outcome phenotypes. In addition, for each region, SKAT analytically calculates a p-value for association.

We also investigated patterns of rare variation in Klassen et al.’s [16] set of 237 genes related to neurotransmission. We filtered our variants that were predicted to alter protein function (i.e., nonsynonymous, stop-gain, stop-loss, frameshift, and splice-junction mutations) and that were present at ≤1% frequency in public databases. To filter out apparent false positive heterozygous calls as judged by a skew in allelic balance, we also applied a lower limit of 1% probability on the ratio of observed reads from the two alleles under a binomial model.

Choice of a pathogenicity predictor. Several pathogenicity predicting methods are available, each of which is based on different methods to assess the impact of a given substitution. Our approach here was to use a statistical procedure to determine the single best pathogenicity predictor in order to avoid issues associated with a multiple testing procedures. In particular, because we assess pathogenicity across 237 genes (see below), we wanted to avoid the complications associated with combining results from multiple predictors and/or the significance corrections required when performing multiple tests. The Grantham score was one of the first implemented to predict the effect of an amino acid substitution [20]. This score takes into account protein properties that correlate best with residue substitution frequencies. Subsequently, we considered a variety of strategies to predict pathogenicity. For example, the phyloP score [21] is derived from a statistical approach based on non-neutral substitution rates on mammalian phylogenies. SIFT [22] is also based on protein conservation. Combined Annotation Dependent Depletion (CADD) [23] uses a machine learning approach to integrate multiple annotations. Using protein features, PolyPhen-2 [24] predicts damaging effects of missense mutation via comparisons of a property of the wild-type allele and the corresponding property of the mutant allele.

Because these pathogenicity predictors were assessed over the entire exome, the quality of their predictions may vary depending on the family of proteins under study. Indeed, from our previous work on Dravet syndrome [15], we found PolyPhen-2 to be the most reliable predictor. To certify this formally, we compare the performance of the other three aforementioned pathogenicity scores against PolyPhen-2 using the area under the curve (AUC) for the receiver-operating characteristic. Our data were taken from a set of individuals with epilepsy whose pathogenic mutations were either in the *SCN1A* (*n* = 122) [25] or *SCN8A* gene (*n* = 111). These were matched against the list of variants in the ExAC database (*n* = 346 and *n* = 248, respectively) (http://exac.broadinstitute.org). Using scores of the cases and controls for these sodium channel mutations, the AUC for PolyPhen-2 is 0.798, while the AUCs for the Grantham score and SIFT algorithms were much lower (< 0.6). For CADD and phyloP, the AUCs were 0.759 and 0.757, respectively (**S1 Fig**) To determine the statistical significance of these differences, we used the DeLong test [26] for paired data sets implemented in the pROC R package (http://expasy.org/tools/pROC/). The *p*-values for one-sided tests are 0.025 and 0.038, respectively. A bootstrap approach produced essentially the same *p*-values. Hence, in the following analyses we use Polyphen-2 to predict whether variants were possibly or probably damaging or benign.

### Power analysis

To estimate our power to distinguish phenotypic classes based upon the number of variants predicted to be benign or damaging, we inferred a reduction in mutational load using a hypothesis-testing framework, estimating the following parameters. The mean number of benign or damaging mutations per individual in a given gene set is μ_0_ for the severely affected and μ_1_ for the mildly affected. For samples of size n_0_ and n_1_, respectively, we model the number of mutations as having a Poisson distribution, means θ_0_ = *n*_0_μ_0_ and θ_1_ = *n*_1_μ_1_. Consequently, statistical power can be determined using the Poisson distribution. As a practical matter, we need to choose a combination of sample size and size of gene set to obtain an estimate of the total number of mutations equal to the desired value of θ_0_. For example, if we want to investigate further the role of GABA genes, choride channel genes, or any small gene set, we would increase the sample size in the study to obtain the desired power.

To illustrate power calculations, we set the sample sizes to be equal in the two groups and the significance level alpha = 0.01. For perfect classification (benign or damaging) and mutational load half as large for the mild group as in the severe group, we require θ_0_ ≥30 to achieve at least 80% power. For mutational load 2/3 as large, we require θ_0_ to be at least 75. For a more realistic analysis, assume 10% false negative (classifying deleterious as benign) and 40% false positives, then the threshold values for θ_0_ rise to 67 and 216 for the two examples above. This substantial increase in sample size is necessary to account for misclassified mutation calls or an improvement in pathogenicity prediction.

## Results

**Table 1** lists key characteristics of the patients in the study. We chose 22 subjects at opposite ends of a phenotypic distribution (11 mild and 11 severe) from a large cohort of Japanese patients with *SCN1A* truncation variants [15]. During the course of the study the two youngest patients in the mild category progressed to a more severe phenotype, leaving 9 individuals in the mild group. One of these patients was then included in the severe group (n = 12). For severe individuals, the mean time between onset of seizures (mean = 5.2±2.3 months) and the initial diagnosis as ‘severe’ is 56.3±27.5 months. For the mild patients, the mean time since seizure onset (7.6±3.0 months) and the most recent assessment as ‘mild’ (i.e., length of time patient remained classified as mild) is 186.1±39.4 months (p-value for difference in seizure onset age = 0.063, t-test, and p = 0.062, Wilcoxon ranked sum test). The 5 males and 4 females in the mild group had IQ values ranging from 50 to 85 (mean = 64.1±9.9); while the 4 males and 8 females in the severe group all had an IQ <24. Eleven of the 12 severe patients experienced a motor delay compared with 3 of the 9 mild patients. The approximate positions of the *de novo* truncation mutations (10 frameshift, 7 nonsense, 2 microdeletion, and 1 splicing) within the Nav1.1 channel are shown **S2 Fig**.

**Table 1:** Characteristics of individuals in study.

Mild group	Sex	Diagnosis	IQ	motor delay	Mutation	exon affected	Seizure onset age (months)	No. months between seizure onset and last assessment
M1	M	DS border	61	N	microdel	1~16	6	261
M2	M	GEFS+	50–75	N	nonsense	16	6	189
M3	F	DS	70–85	N	nonsense	1	15	180
M4	F	DS	50–70	N	splicing	intron 5	5	190
M5	M	DS	59	Y	frameshift	16	8	187
M6	F	FS+	66	N	frameshift	23	8	187
M7	F	DS	65	Y	frameshift	16	7	188
M8	M	DS	50	Y	frameshift	16	6	189
M9	M	DS border	81	N	nonsense	2	7	104
Severe group								No. months between seizure onset and severe diagnosis
S1	F	DS	<24	N	frameshift	20	6	44
S2	M	DS	<24	Y	frameshift	10	4	39
S3	F	DS	<24	Y	frameshift	2	6	43
S4	F	DS	<24	Y	frameshift	1	6	41
S5	M	DS	<24	Y	nonsense	5	9	48
S6	F	DS	<24	Y	frameshift	11	5	45
S7	M	Epilepsy/ID	<24	Y	microdel	1~26	2	59
S8	F	DS	<24	Y	frameshift	10	6	61
S9	M	DS	<24	Y	nonsense	19	2	40
S10	F	DS	<24	Y	nonsense	24	8	47
S11	F	DS	<24	Y	nonsense	17	2	70
S12	F	DS	<24	Y	microdel	1~26	6	138

DS, Dravet syndrome; GEFS+, genetic epilepsy with febrile seizures plus; F, female; M, male.

**Fig 1:**
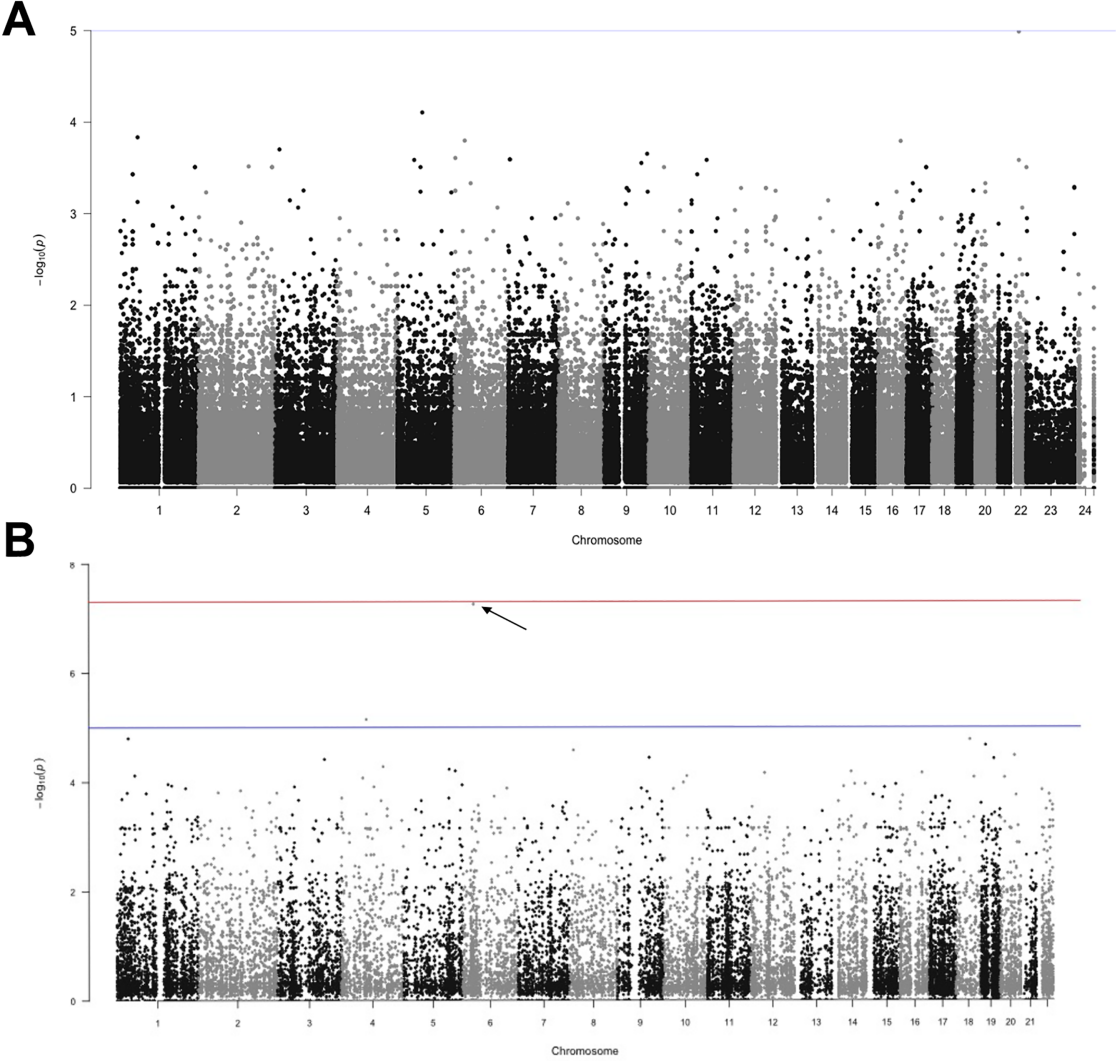
Manhattan plot of p-values. (A) Fisher exact tests performed on genotype counts for common variants identified by whole exome sequencing in the mild *versus* severe groups. (B) Association tests based on aggregated sets of both common and rare contiguous SNPs across the exonic regions of the genome. P-value associated with *FKBP5* is indicated with arrow.

Our analysis of the exome sequences uncovered all of the truncating variants that were originally identified by PCR and Sanger sequencing in our cohort of 22 individuals, except for the three cases of microdeletions covering large sections of (or the entire) *SCN1A* gene (**S2 Table**). Considering all other variants in the complete dataset and after filtering for quality, we asked whether particular variants are enriched in either the mild or severe phenotypic groups. We did this by performing Fisher exact tests on genotype counts in the two groups. **Fig 1A** shows a Manhattan plot of p-values, the smallest of which is associated with an intronic variant in the eye lens gene, *CRYBA4* (p = 1.02 x 10^−5^). We note that the smallest possible p-value given our sample sizes is 3.4 x 10^−6^ (i.e., the case of fixation of opposite genotypes in the two phenotypic groups). The smallest p-value associated with a non-synonymous variant (*ZNF615*, p = 5.6 x 10^−4^), ranked 40^th^ in the list of smallest p-values (**Fig 1A**). Of the top 40 variants, only 4 were exonic (HYDIN, MACF1, ETNK1 and ZNF615), and only the ZNF615 gene is expressed in the brain (**S3 Table**).

**Fig 1B** shows results of the gene-based association test, in which we find that gene region *FKBP5* on chromosome 6 shows a highly statistically significant association with phenotypic class (p = 6.2 x 10^−7^). There are 13 variants in this region in our exome data, 5 of which are common SNPs (MAF ≥5%). Importantly, statistical significance was greatly reduced when we did not include gender as a covariate (p = 1.7 x 10^−3^), suggesting that sex is an important driver of this association. We then attempted to adjust covariates to account for potential factors that can bias SNP effect estimates and to improve statistical power by reducing residual variance [27]. Given the correlation between severity (mild and severe) and motor delay is 0.612 (Pearson and Spearman), we first wanted to ask whether the signal was from one or both of these covariates. When we switched primary outcome to motor delay and severity to a covariate, *FKBP5* does not show a statistically significant association (p = 0.022). Therefore we excluded the possibility that the signal from FKBP5 was due to bias from adjusting covariates.

In an effort to detect the causal SNP, we performed a logistic regression using the same four covariates and computed the contribution of each SNP to the test statistic following the methods of Lee et al. [28]. The total score statistic using equal weights is 336.7 (mean = 25.9 ± 32.5), and the partial scores for each SNP showed that nearly half of the signal comes from two SNPS: rs9348979 and rs79549155 (**S4 Table**). For these two SNPs, only a single subject in our study carried the minor allele. Moreover, this particular individual in the mild group (M7, **Table 1**) has a large residual (-8.84). When we remove M7 from the analysis, the total score statistic is reduced to 148.4 (mean = 12.4 ± 15.6). This result suggests that our finding of an association between *FKBP5* and clinical severity may be biased by inclusion of a single female individual in the mild group.

We genotyped the intronic *FKBP5* T>C polymorphism at rs1360780, which has been identified in GWAS as risk allele for stress-related disorders [29, 30] (**S5 Table**). We find a similar frequency of the three genotypes in the mild and severe groups; however, we note that mild males have a higher frequency of the T/T genotype (0.40) compared with mild females (0.0). Severe males (0.25) and females (0.13) have intermediate frequencies of the T/T genotype. If we compare our data with 1000 Genomes East Asian genotype data [17] at this SNP (frequency = 0.05, n = 504), we find a statistically significantly higher frequency of the T/T genotype (p = 0.026; two-tailed Fisher exact test) in our combined DS sample (frequency = 0.19, n = 21).

**Fig 2:**
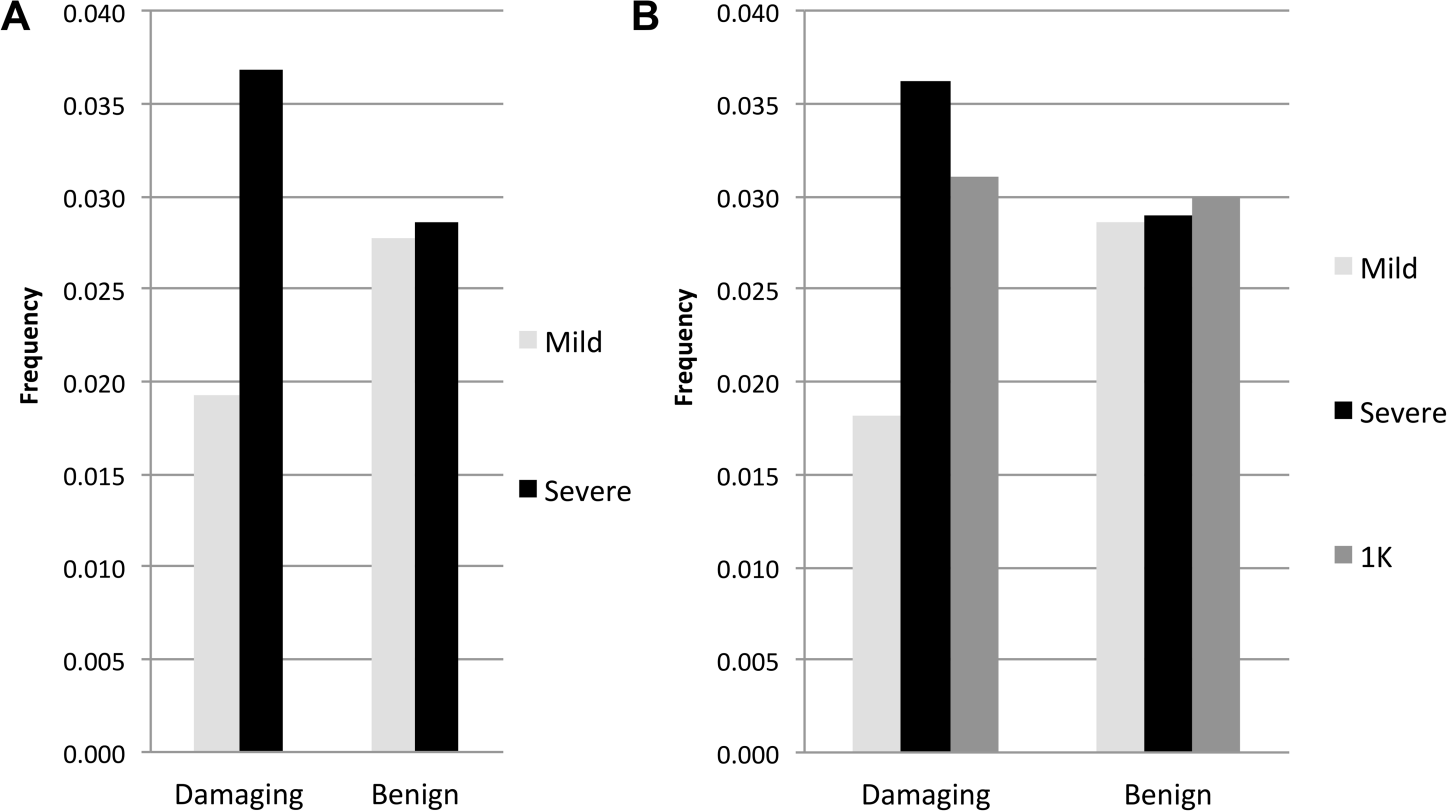
Frequency of rare missense variants predicted to be damaging and benign in 237 neuronal excitability genes. (A) Mild *versus* severe group. (B) Mild, severe and 1000k genomes East Asian exomes. Frequencies were calculated by dividing the number of alternate alleles by the total number of alleles in each class.

We screened the set of candidate genes associated with neurological excitability produced by Klassen et al. [16] to search for rare, missense variants in our exome data. After filtering for high quality variants, we limited our search to missense variants that were ≤1% in the ExAC browser and then used PolyPhen-2 to predict whether these missense variants were damaging or benign. Within the 237 genes we found 122 missense variants in our cohort of 21 individuals, 52 and 70 of which were predicted to be damaging or benign, respectively (**Table 2**). We then asked whether rare damaging missense variants are present at lower frequency in the mild *versus* severe groups. The 9 mildly affected individuals carry a total of 18 alleles predicted to be damaging, while the 12 severely affected individuals carry a total of 46 (**Table 2**). We note that all variants were singletons (i.e., present in only 1 copy among the 21 individuals), except in the case of five variants among severely affected individuals, whereby two individuals shared a variant at the *HTR1A*, *KCNJ1*, *KCNJ14* and *CACN1B* loci, while three individuals shared the same variant (p.E515D) at the *KCNQ2* locus (**S6 Table**). When we compare the number of damaging alleles relative to the number of reference alleles between the two phenotypic groups (i.e., 18 *versus* 918 and 46 *versus* 1202, respectively), we find a statistically significant excess of damaging alleles in the severe group (p = 0.0100, one-tailed Fisher exact test). This result contrasts with the distribution of benign missense alleles, which are found in approximately equal frequencies in the mild (35 *versus* 1225) and severe (48 *versus* 1632) groups (p = 0.9111, two-tailed Fisher exact test). The frequency of damaging and benign alleles in mildly and severely affected individuals is shown in **Fig 2A**.

**Table 2:** Rare missense alleles predicted to be damaging and benign in 237 neuronal excitability genes.

		Damaging		Benign	
Gene Class	# Genes	Mild	Severe	Mild	Severe
Cholinergic Receptor Genes	16	3	4	2	3
Dopamine Receptor Genes	5	0	0	2	2
GABA Receptor Genes	20	0	5	3	5
Glycine Receptor Genes	9	0	0	1	0
Ionotropic Glutamate Receptor Genes	14	0	3	1	1
Metabotropic Glutamate Receptor Genes	8	1	3	1	1
Serotonin Receptor Genes	16	3	3	3	3
Voltage-gated Calcium Channel Genes	26	0	6	3	8
Chloride Channel Genes	9	2	1	4	4
Voltage-gated Potassium Channel Genes	50	5	9	5	5
Voltage-gated Sodium Channel Genes	14	1	2	4	7
Calcium Activated Potassium Channel Genes	8	0	0	0	2
Potassium Inwardly Rectifiying Channel Genes	14	2	4	1	0
Twin Pore Potassium Channel Genes	14	0	0	1	1
Cyclic Nucleotide-gated Channel Genes	4	0	1	0	1
Ryanodine Receptor Genes	3	1	4	4	3
Other	7	0	1	0	2

To compare the prevalence of rare damaging variants in our DS cohort with that in healthy populations, we accessed the BAM files for 70 JPT exomes made public by the 1000 Genomes project [17]. To control for different sample sizes and variant calling methods we processed the 22 public samples with the fewest missing sites in the 237 genes analyzed above along with our exome data in a single variant calling analysis. As a result of quality differences between data sets, we were not able to call all of the same variants that were called when analyzing our data separately. Within the 237 genes we found 118 missense variants in our cohort of 21 DS individuals, 46 and 72 of which were predicted to be damaging or benign, respectively (**Table 2**). There are 15 and 40 alleles predicted to be damaging in the mildly and severely affected individuals, respectively; which again results in a statistically significant excess of damaging alleles in the severe group (p = 0.0116, one-tailed Fisher exact test).

We find 160 alleles in the public data, of which 56 and 104 are predicted to be damaging and benign, respectively. **Fig 2B** plots the frequency of damaging and benign alleles in our DS cohort and in the public JPT exomes. Unlike the case in mildly *versus* severely affected individuals in our DS cohort, we find a similar frequency of damaging (56/1804 = 0.031) and benign (104/3476 = 0.030) alleles in the public data. Moreover, we note that healthy individuals have an intermediate frequency of damaging alleles (0.031) relative to the frequencies we find in mildly (0.019) and severely (0.037) affected DS individuals, while all three groups have similar frequencies of benign alleles (0.029, 0.029, 0.030, respectively).

## Discussion

An important goal in human genetics is to identify genetic factors that modulate the clinical expression of monogenic diseases. Characterization of modifier genes not only leads to a better understanding of syndrome-specific pathophysiology, it also helps to facilitate novel therapeutic approaches. Much of our current knowledge of the pathophysiology of epilepsy comes from animal models, many of which were constructed with genetic variants discovered in humans. Indeed, the observation that phenotype severity in Scn1a^+/−^ mice was strongly dependent on strain background led to the discovery of modifiers in mouse models of DS [3, 31]. Identifying modifier genes in humans has proven to be much more difficult. Anecdotal evidence has pointed to variants in ion channels as modifiers of the severity of the DS phenotype. Singh et al. [32] suggested that variants in the closely linked *SCN9A* gene were associated with increased severity of the disorder, while Ohmori et al. [33] suggested the same for rare variants in the *CACNA1A* gene. On the other hand, Gaily et al. [34] proposed that variants in *POLG* (a catalytic subunit of mitochondrial DNA polymerase) increase susceptibility for acute encephalopathy in DS.

To perform a more systematic search for genetic variants affecting the severity of DS, we sequenced the exomes of 22 Japanese patients with *SCN1A* truncation variants and whose clinical outcomes represented opposite ends of a phenotypic distribution. We chose to restrict our study to epilepsy patients carrying a truncation mutation in *SCN1A* to control for the effect of ‘mutation type’ on clinical outcome. Truncation mutations anywhere in the *SCN1A* gene other than in exon 26 are predicted to result in nonsense mediated RNA decay and haploinsufficiency [15]. Unlike missense variants that are present in different functional domains of the channel and that result in different amino acid substitutions, truncation mutations can be considered a single “genetic phenotype.” Barring the effects of other factors, truncation mutations are expected to be associated with a more homogeneous clinical outcome [15]. We also chose to control for population structure by considering only a single ethnic group. Given that variants in modifier genes may have little to no effect on the phenotype of individuals that do not carry a pathogenic variant in a ‘driver’ gene, we reasoned that modifier variants could be present as common polymorphisms. By restricting our study to Japanese patients, we had hoped to minimize the possibility of a loss of power due to the segregation of more than a single common modifier in the sample. A liability of this approach is that our sample size was limited by the rarity of patients with haploinsufficiency and mild clinical symptoms.

The first test attempted to identify SNP genotypes that differed in frequency in mildly affected *versus* severely affected individuals. Although we had low power, no single genotype emerged from this analysis, and those that showed the greatest differentiation between phenotypic groups were variants in non-coding regions of the exome. The second test sought to boost power by aggregating sets of contiguous SNPs, both common and rare, and by searching for a phenotypic association across the exonic regions of the genome. Interestingly, 13 SNPs positioned across the *FKBP5* locus together produced a statistically significant association when clinical severity was selected as the outcome and gender as a covariate. This association disappeared when gender was ignored, suggesting that sex-associated factors played a role in this association. *FKBP5* is a co-chaparone of the glucocorticoid receptor and participates in regulation of glucocorticoid receptor sensitivity, and variants at this locus have been associated with risk of post-traumatic psychological sequelae and post-traumatic pain outcome [35, 36]. The intronic SNP rs1360780 in this gene (T allele *versus* C homozygous) has been shown to be associated with increased *FKBP5* protein levels and prolonged stress hormone activation following a stressor [37]. Stress-induced activation of the hypothalamic-pituitary-adrenal (HPA) axis leads to compromised GABAergic inhibition in the hippocampus, and to increased neuronal excitability and seizure susceptibility [38, 39]. Interestingly, sex-dependent differences in the HPA axis stress response are believed to contribute to the different prevalence rates of stress-related disorders in females and males [40]. We found an increased frequency of the rs1360780-T allele in our DS cohort relative to an East Asian population, which may be accounted for by an excess of this allele in males with a mild outcome. However, we also found that statistical significance was lost when gene-based association analysis was performed after removing a single female in our cohort, suggesting that that this association may be a false positive. Further work with a much greater sample size is needed to confirm an association between variation at *FKBP5* and clinical outcome in DS.

In our final test, we searched a set of 237 neurotransmission genes [16] for rare variants (MAF≤1%) in the exome sequences of our epilepsy cohort. Interestingly, we observe a greater frequency of rare damaging alleles in the severely affected group, while benign alleles show a similar frequency in mildly and severely affected groups (**Fig 2A**). The gene classes with the lowest ratio of damaging alleles between the mild and severe groups (relative to that for benign alleles) are the GABA receptors and voltage-gated calcium channels. When Klassen et al. [16] compared exonic region variant profiles for this set of 237 ion channelopathy candidate genes in a cohort of unaffected individuals and those with sporadic idiopathic epilepsy, they observed overlapping patterns of both rare and common variants across both populations. They suggested that deleterious ion channel mutations confer uncertain risk to an individual depending on the other variants with which they are combined, thus revealing a complex allelic architecture underlying personal disease risk. Our results parallel those of Klassen et al. [16] in that we do not find a single modifier influencing outcome in *SCN1A*-truncation positive DS; rather we find that the constellation of rare variants in an individual’s genetic background can tip the balance toward a mild or severe outcome when coupled with *SCN1A* haploinsufficiency. Individually, each rare damaging variant may not have a clinically significant effect on healthy individuals. Indeed healthy individuals appear to carry nearly as many damaging rare variants as severely affected patients with truncation mutations in *SCN1A* (**Fig 2B**). This is consistent with hypothesis that rare pathogenic variants of small effect size in genes associated with neuronal excitability may tip the balance toward mild or severe clinical outcome.

A key difference between studies is that Klassen et al. [16] searched for variants in the gene(s) responsible for epilepsy, while we searched for modifiers of clinical variability in individuals with a primary variant known to cause DS. Interestingly, there are no known modifiers that completely protect individuals with *SCN1A* truncation mutations, and no *SCN1A* truncation variants are present in large public databases of healthy individuals (i.e., without childhood epilepsy). Because all patients with DS tend to progress from a mild to a severe cognitive phenotype [15], the difference between our phenotypic groups—and hence what putative modifiers may be acting upon—are processes that influence the rate of cognitive decline.

In the Scn1a^+/-^ DS mouse model, Hawkins et al. [2] recently performed expression analysis and identified the GABA_A_ receptor subunit, Gabra2, as a putative modifier gene. They suggested that neurotransmitter receptors modulate the effects of loss of function mutations at Scna1 by influencing the excitatory/inhibitory balance. Variation at other ion channel genes has been suggested to modify spontaneous seizure activity and the lifespans of Scn1a^+/-^ mutants [1, 12, 13, 41]. We note that a single variant predicted to be damaging in the potassium channel gene, *KCNQ2*-p.E515D, was found in three of our patients with severe DS (S2, S3 and S8). This variant has been reported in several families with benign neonatal seizures (BFNE) and is thought to be a risk allele for epilepsy [42]. There is no evidence that these three patients exhibited signs of BFNE, which usually presents with seizures in the first week of like (e.g., mean seizure onset was 5.3 ± 1.2 months, very similar to the mean of 5.1 ± 2.6 for the other 8 severely affected individuals). However, it is interesting to note that patients with the *KCNQ2*-E515D variant may exhibit a higher rate of intellectual disability than BFNE patients with other *KCNQ2* variants (Lee et al. 2016). Our patients with *KCNQ2*-p.E515D also differ with respect to typical EEG and seizure semiology patterns observed in *KCNQ2* encephalopathy. For example, EEGs often show a suppression burst pattern in *KCNQ2* encephalopathy, whereas our patients either had normal EEGs at seizure onset (S2 and S8) or showed hemisphere dominant epileptic discharges (S3). While *KCNQ2* encephalopathy is often associated with epileptic spasms at onset, our patients exhibited hemiconvulsions (S2), generalized tonic clonic convulsions (GTCC) triggered by fever (S3), and myoclonus of upper limbs and secondary GTCC (S8), and went on to develop generalized tonic seizures (S2 and S3) or seizure freedom (S8).

Our finding of a similar frequency of damaging alleles among both healthy individuals and severely affected individuals in the same set of neuronal excitability genes suggests that the mildly affected patients have a reduced mutational load. This makes sense in light of the fact that it is extremely difficult to identify truncation-positive DS patients with a persistently mild phenotype. Therefore, by culling such individuals from the population at large we may have unintentionally selected for outliers with fewer damaging rare alleles at these loci. It is tempting to speculate that that the penetrance of other monogenic disorders may be influenced by the high prevalence of rare damaging alleles segregating at multiple loci in the human population. Indeed, the recent explosive growth experienced by human populations has been implicated in increasing the load of deleterious rare variants, which now play a role in the individual genetic burden of complex disease risk [43, 44]. We suggest that this process also may have influenced current clinical outcome in monogenic (oligogenic) disease.

## Supporting information

S1 Fig Receiver operating characteristic for the pathogenicity prediction scores, PolyPhen-2, phyloP, and CADD for binary classification of benign *versus* pathogenic for *SCN1A* and *SCN8A* variants. Benign variants were taken from the ExAC database and pathogenic variants from Ishii et al. [15].
(TIF)

S2 Fig Approximate positions of the *de novo* truncation mutations (10 frameshift, 7 nonsense, 2 microdeletion, and 1 splicing) within the Na_V_1.1 channel. (TIFF)

S1 Table List of 237 candidate genes associated with neurological excitability in Klassen et al. (XLSX)

S2 Table Truncating variants identified by WES in our cohort of 22 individuals (originally identified by multiplex ligation-dependent probe amplification (MLPA) and/or PCR-Sanger sequencing). (XLSX)

S3 Table Top 40 variants in Test 1. (XLSX)

S4 Table *SKAT* test statistic for *FKBP5* SNPs. (XLSX)

S5 Table *FKBP5* functional SNP genotypes. (XLSX)

S6 Table Neuronal excitability gene variant genotypes. (XLSX)
