# Supplementary figures and images for: Rare variants of small effect size in neuronal excitability genes influence clinical outcome in Japanese cases of SCN1A truncation-positive Dravet syndrome
Source: PLoS One. 2017 Jul 7;12(7):e0180485. doi: 10.1371/journal.pone.0180485 (PMC5501540; doi:10.1371/journal.pone.0180485)

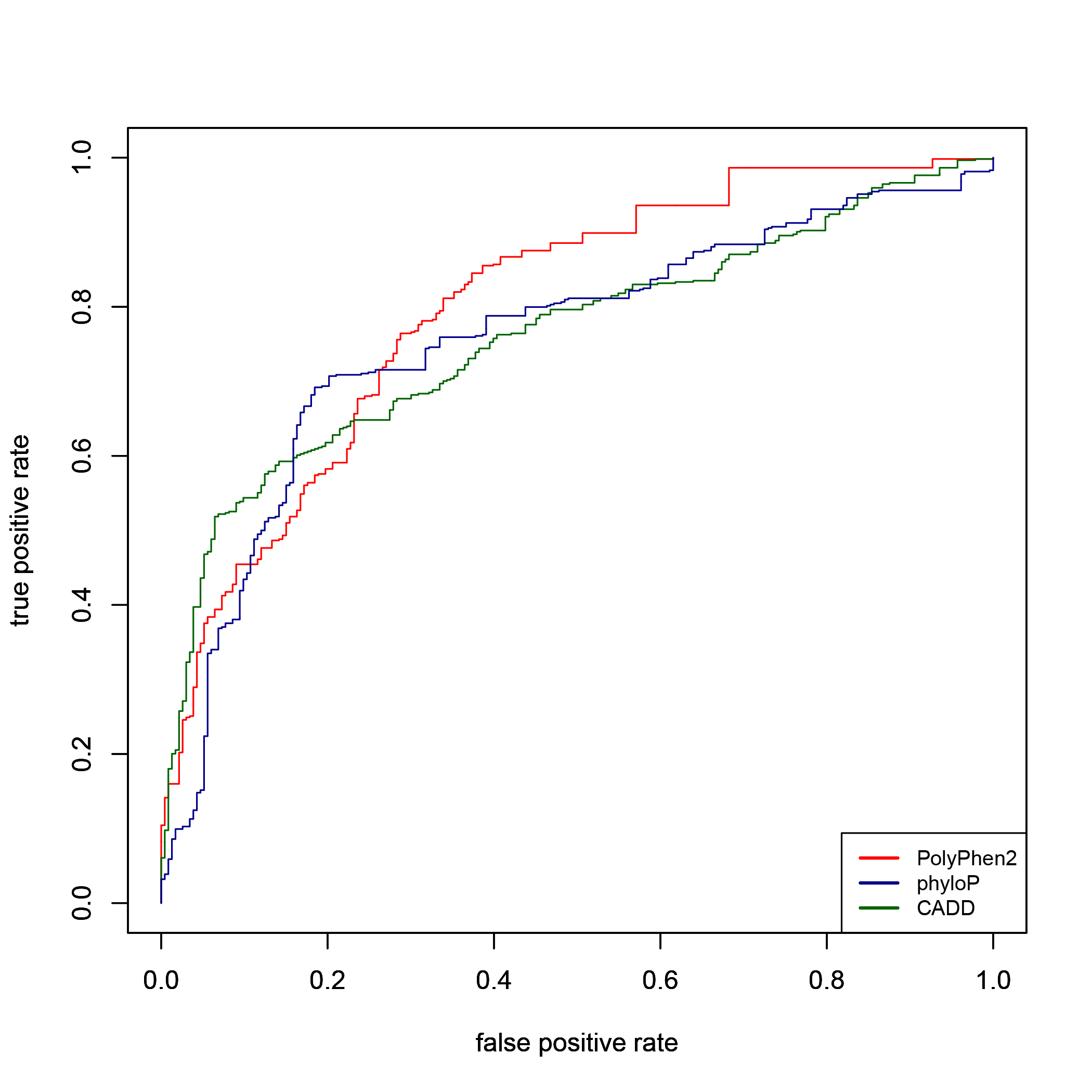

Supplement: S1 Fig — Benign variants were taken from the ExAC database and pathogenic variants from Ishii et al. [15]. (TIF) [file pone.0180485.s001.tif]

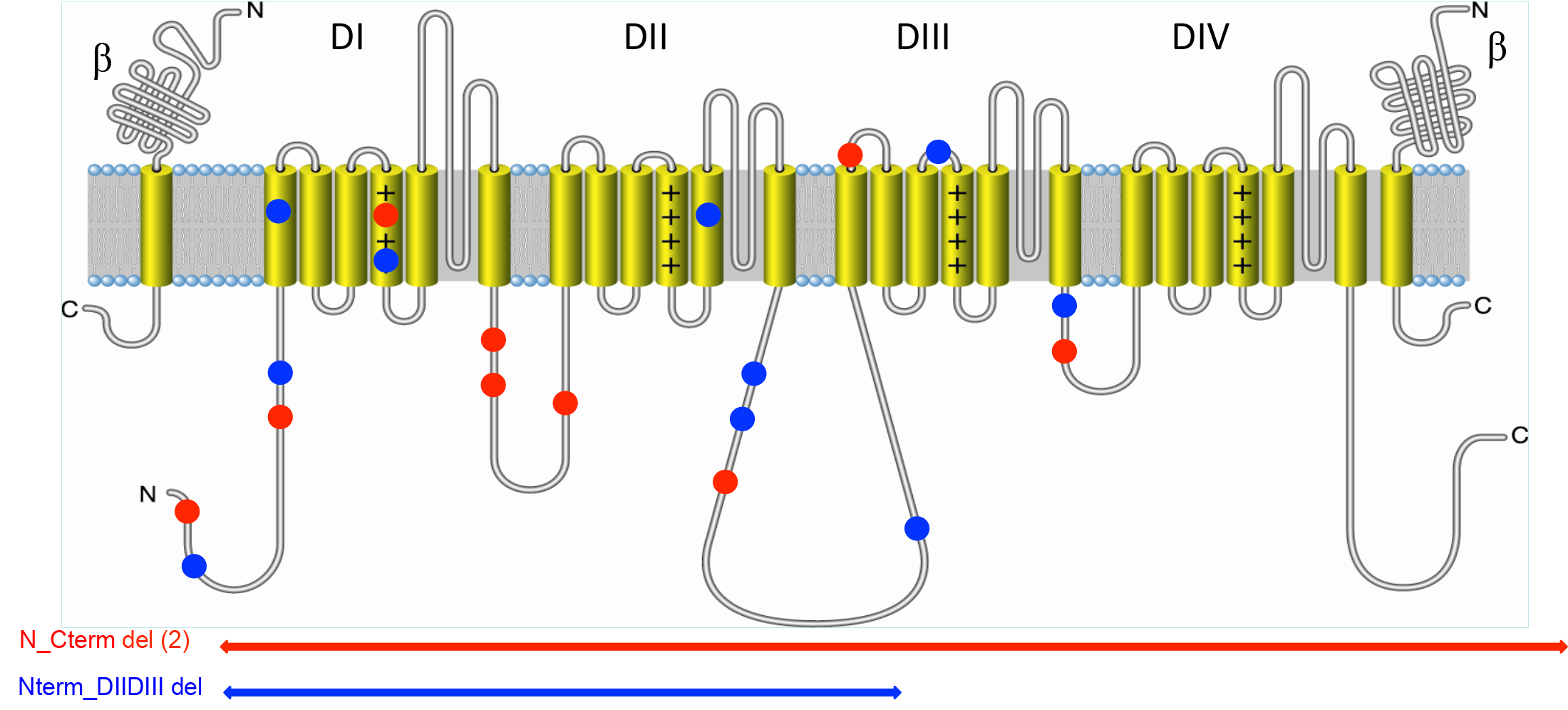

Supplement: S2 Fig — (TIFF) [file pone.0180485.s002.tiff]
